# Supplementary material for: Effect of Moderate Exercise on the Superficial Zone of Articular Cartilage in Age-Related Osteoarthritis
Source: Diagnostics (Basel). 2023 Oct 12;13(20):3193. doi: 10.3390/diagnostics13203193 (PMC10605492; doi:10.3390/diagnostics13203193)
Supplement: Supplementary file 1 [file diagnostics-13-03193-s001.zip › diagnostics-2631954-supplementary.pdf]

Supplementary Materials:

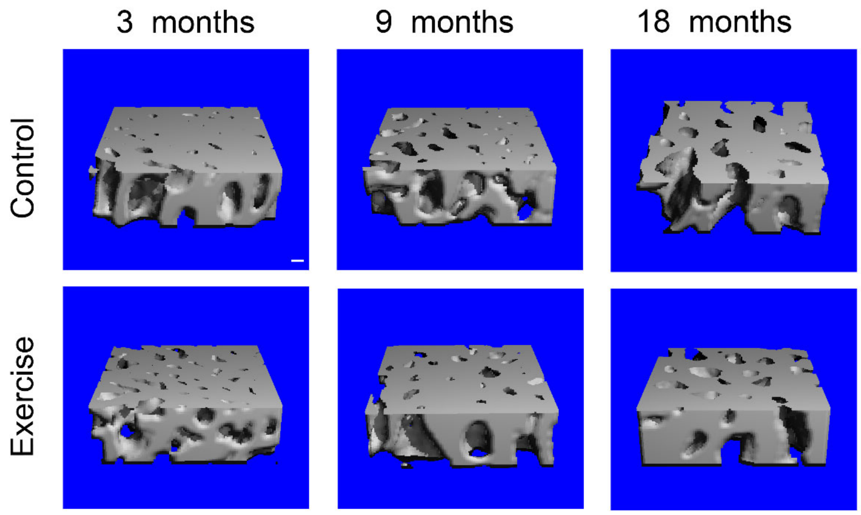

Figure S1: Representative cubic regions of interest (0.56 × 1.5 ×1.5 mm^3) were selected from the subchondral bone of the tibial plateau.

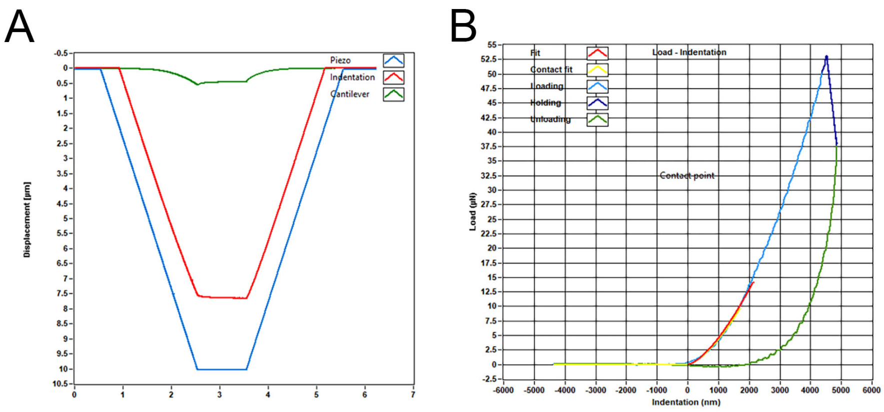

Figure S2: (A) The load-indentation curve ensured the reliability of tests. (B) Pressing on the cartilage surface under liquid conditions.

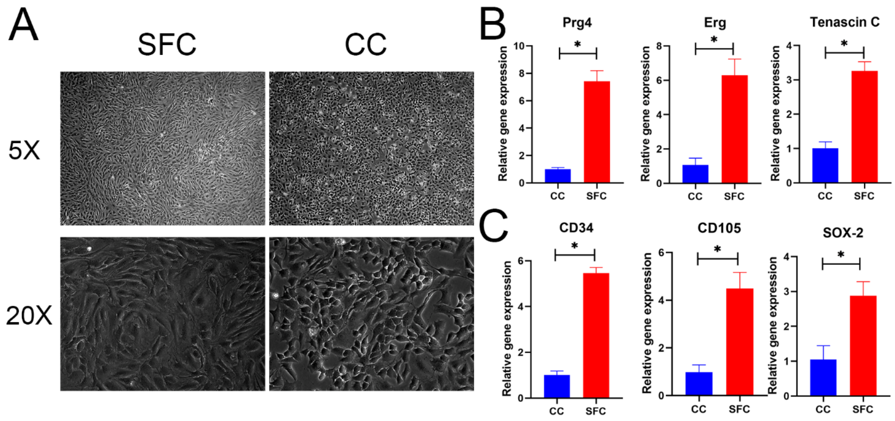

Figure S3: Isolation and culture of SFZ cells and related gene expression analysis: (A) The SFZ cells and chondrocytes (CC) were observed using a fluorescence microscope. Total RNAs were prepared from SFZ or chondrocyte confluent cultures and subjected to quantitative PCR analysis for SFZ markers (B: Prg4, Erg and Tenascin C), and stem cell markers (C: CD34, CD105 and Sox2).
